# Prediction of Multiple-Trait and Multiple-Environment Genomic Data Using Recommender Systems

**DOI:** 10.1534/g3.117.300309

**Published:** 2018-01-04

**Authors:** Osval A. Montesinos-López, Abelardo Montesinos-López, José Crossa, José C. Montesinos-López, David Mota-Sanchez, Fermín Estrada-González, Jussi Gillberg, Ravi Singh, Suchismita Mondal, Philomin Juliana

**Affiliations:** * Facultad de Telemática, Universidad de Colima, 28040 Colima, México; † Departamento de Matemáticas, Centro Universitario de Ciencias Exactas e Ingenierías (CUCEI), Universidad de Guadalajara, 44430 Jalisco, México; ‡ International Maize and Wheat Improvement Center (CIMMYT), Apdo. Postal 6-641, 06600 México City, México; § Departamento de Estadística, Centro de Investigación en Matemáticas (CIMAT), 36240 Guanajuato, México; ** Department of Entomology, Michigan State University, East Lancing, Michigan 48824; †† Department of Computer Science, Aalto University, FI-00076, Finland

**Keywords:** genomic information, item-based collaborative filtering, matrix factorization, multi-trait, genotype, environment interaction, prediction accuracy, collaborative filtering, GenPred, Shared Data Resources, Genomic Selection

## Abstract

In genomic-enabled prediction, the task of improving the accuracy of the prediction of lines in environments is difficult because the available information is generally sparse and usually has low correlations between traits. In current genomic selection, although researchers have a large amount of information and appropriate statistical models to process it, there is still limited computing efficiency to do so. Although some statistical models are usually mathematically elegant, many of them are also computationally inefficient, and they are impractical for many traits, lines, environments, and years because they need to sample from huge normal multivariate distributions. For these reasons, this study explores two recommender systems: item-based collaborative filtering (IBCF) and the matrix factorization algorithm (MF) in the context of multiple traits and multiple environments. The IBCF and MF methods were compared with two conventional methods on simulated and real data. Results of the simulated and real data sets show that the IBCF technique was slightly better in terms of prediction accuracy than the two conventional methods and the MF method when the correlation was moderately high. The IBCF technique is very attractive because it produces good predictions when there is high correlation between items (environment–trait combinations) and its implementation is computationally feasible, which can be useful for plant breeders who deal with very large data sets.

Plant breeding programs aim to improve multiple traits and develop new, higher yielding varieties that are disease and drought resistant and regionally adapted to different environments and growing conditions. However, to reach that challenging goal, plant breeders have been looking for new tools to improve the selection of candidate genotypes in early stages. Genomic selection (GS) proposed by Meuwissen *et al.* (2001) is one of the most powerful tools used by breeders and is now revolutionizing plant breeding. One of the fundamental features of GS is the use of high-density markers. Rather than seeking to identify individual loci that are significantly associated with a trait, GS uses all marker data simultaneously as performance predictors of a line, and this consequently delivers predictions that are more accurate. Selection under this approach is based on GS predictions, potentially leading to more rapid genetic gains at a lower cost. Also, GS allows marker data to be combined with phenotypic and pedigree data (when available) in an attempt to increase the accuracy of the prediction of breeding and genotypic values (Goddard and Hayes 2007).

Although there is empirical evidence showing that GS is a powerful tool for selecting candidate genotypes in early stages, there is still the need for novel statistical models for selecting multiple correlated traits evaluated in multiple environments. Recent examples of publications on this type of models for multiple traits and multiple environments are those of Montesinos-López *et al.* (2016, 2017) for continuous and count data. However, although these proposed models are mathematically elegant, they are computational very limited; as the number of genotypes, environments, and traits increases, these models cannot be implemented because it is extremely difficult to sample from large multivariate normal distributions. For the abovementioned reasons, statisticians and plant scientists are open and looking for better prediction alternatives in the context of data for multiple traits and multiple environments. However, the need to increase prediction accuracy is not exclusive to GS; it is present in many other areas like marketing, e-commerce, finance, and biology. Each area has made a significant effort to increase predictions; however, in general, few cases have been successful. This reminds us that making predictions is usually very challenging since the target inference is to predict unobserved quantities at the time of the inferences.

In this article, we will explore two methods that are very popular in recommender systems. A recommender system is a subclass of an information filtering system that seeks to predict the “rating” or “preference” that a user would give to an item (Ricci *et al.* 2011). Recommender systems have become increasingly popular in recent years, and are utilized in a variety of areas including movies, music, news, books, research articles, search queries, social tags, and products in general. In this research, we will implement the following two recommender systems methods in the context of GS: item-based collaborative filtering (IBCF) and the matrix factorization method. The first method (IBCF) belongs to the collaborative filtering techniques, which are a set of prediction methods that have been significantly successful in building recommender systems in various settings (*e.g.*, marketing, e-commerce, *etc*.). The basis of this technique is to use the known preferences of a group of users to make recommendations or predictions of the unknown preferences of other users.

The second method (matrix factorization) belongs to the latent factor models, and tries to explain the ratings by characterizing both items and users on a reduced number of factors that approximate the rating matrix. The matrix factorization method is considered one of the most successful of the latent factor models. In its basic form, matrix factorization characterizes both items and users by vectors of factors inferred from item rating patterns. High correspondence between item and user factors leads to a recommendation. These methods have become popular in recent years by combining good scalability with predictive accuracy. In addition, they offer much flexibility for modeling various real-life situations.

The two recommender systems mentioned above (IBCF and matrix factorization) work by building a matrix of preferences (called a rating matrix) where each row represents a user, each column represents an item, and the number at the intersection of a row and a column represents the user’s rating value. The absence of a rating score at this intersection indicates that that user has not yet rated the item. We will use simulated and real data to compute the prediction accuracy of both recommender systems (IBCF and matrix factorization) and we will compare their predictions with those produced by two conventional GS models that take into account the genotype × environment interaction term.

## Materials and Methods

### IBCF

As mentioned above, the IBCF technique is a model-based algorithm for recommender items or products. In IBCF, similarities between items are calculated from a rating matrix (Table 1); based upon those similarities, a user’s preference for an item that has not been rated by a user is calculated. In general, Table 1 can be constructed for n users and m items. Here, we present a step-by-step example of IBCF with four users and three items. For example, let us assume that we have the rating matrix shown in Table 1. With this information, we create an item-to-item similarity matrix (using the cosine similarity $=\cos\left(\theta \right)=\sum_{j=1}^{n}x_{j}y_{j}/\sqrt{\sum_{j=1}^{n}x_{j}^{2}}\sqrt{\sum_{j=1}^{n}y_{j}^{2}}$ or Pearson correlation), which provides information on how similar an item is to another item. The complete cosine similarity matrix between items for this example is shown in Table 2.

**Table 1:** Rating matrix data set with four users (rows) and three items (columns), with the rating of each user given in an ordinal scale of three points

User/Items	I1	I2	I3
U1	$y_{1,1}=$ 2	$y_{1,2}=$ ?	$y_{1,3}=$ 3
U2	$y_{2,1}=$ 5	$y_{2,2}=$ 2	$y_{2,3}=$ ?
U3	$y_{3,1}=$ 3	$y_{3,2}=$ 3	$y_{3,3}=$ 1
U4	$y_{4,1}=$ ?	$y_{4,2}=$ 2	$y_{4,3}=$ 2

? denotes missing values that need to be predicted.

**Table 2:** Item-to-item similarity matrix constructed with information in Table 1

	I1	I2	I3
I1	$w_{1,1}$ = 1	$w_{1,2}$ = 0.76	$w_{1,3}$ = 0.78
I2	$w_{2,1}$ = 0.76	$w_{2,2}$ = 1	$w_{2,3}$ = 0.86
I3	$w_{3,1}$ = 0.78	$w_{3,2}$ = 0.86	$w_{3,3}$ = 1

Value$\;w_{j,j´}$ represents the similarity between item *j* and item *j*´, obtained using cosine similarity.

We then predict each user’s ratings for items that have not previously been rated. In this example, we will calculate the rating for user u1 in item I2, along with user u2 in item I3 and for user u4 in item I1. Each of these predictions can be calculated using a simple weighted average to predict the rating, $P_{i,j´},$ for user i in item j´, as follows (Sarwar *et al.* 2001):

$$P_{i,j´}=\frac{\sum_{j\epsilon N}y_{i,j}w_{j,j´}}{\sum_{j\epsilon N}\left|w_{j,j´}\right|},$$ (1)

where the summation is over all other rated items (jϵN) for user i, $w_{j,j´}$ is the weight between items j and j´, and $y_{i,j}$ is the rating for user i on item j. Therefore, the predicted rating for item I2 for user u1 will be $P_{1,2}=\left(y_{1,1}w_{1,2}+y_{1,3}w_{3,2}\right)/\left(|w_{1,2}\right|+\left|w_{3,2}|\right)=\left(2\times 0.76+3\times 0.86\right)/\left(0.76+0.86\right)=2.53,$ while the predicted rating for item I3 for user u2 will be $P_{2,3}=\left(y_{2,1}w_{1,3}+y_{2,2}w_{2,3}\right)/\left(|w_{1,3}\right|+\left|w_{2,3}|\right)=\left(5\times 0.78+2\times 0.86\right)/\left(0.78+0.86\right)=3.43,$ and the predicted rating for item I1 for user u4 will be $P_{4,1}=\left(y_{4,2}w_{2,1}+y_{4,3}w_{3,1}\right)/\left(|w_{2,1}\right|+\left|w_{3,1}|\right)=\left(2\times 0.76+2\times 0.78\right)/\left(0.76+0.78\right)=2.$ We provide the R code for the IBCF in Appendix A1.

It is important to point out that when calculating the similarities between users instead of between items, based upon these similarities, a user’s preference for an item that he/she has not previously rated is calculated. This method is called user-based collaborative filtering (UBCF) and is basically the same as IBCF but refers to users (lines in plant breeding). While item-based algorithms generate predictions based on similarities between items, user-based algorithms generate predictions based on similarities between users. In this article, the main focus is on the IBCF because it is more computationally efficient than UBCF when the number of items is lower than the number of users. Additionally, there is empirical evidence showing that IBCF is more accurate in predicting ratings than UBCF (Sarwar *et al.* 2001).

### Matrix factorization (MF)

Matrix factorization is another algorithm for recommending items or products. Matrix factorization consists of factorizing a matrix, *i.e.*, to find two matrices, which, when multiplied, will produce the original matrix. Formally, the matrix factorization model looks for two matrices, P and Q, of order n×K and m×K, respectively, such that

$$R\approx PQ^{T}=\hat{R},$$ (2)

where R is the matrix that contains all the ratings that the users have assigned to the items and it is of order n×m, K is the prespecified number of latent features (variables) that is lower than or equal to the min(n,m). $\hat{R}$ is the estimated matrix of ratings. Each row of P represents the strength of the associations between a user and the features, while each row of Q represents the strength of the associations between an item and the features. To obtain the prediction of a rating of item $I_{j}\;$by user $u_{i},$ we calculate the dot product of the two vectors corresponding to $u_{i}$ and $I_{j}$:

$${\hat{r}}_{ij}=p_{i}^{T}q_{j}=\sum_{k=1}^{K}p_{ik}q_{kj}.$$ (3)

However, to obtain the predictions for users of the missing items, we need to estimate matrices P and Q, with the no missing entries of the rating matrix. There are several methods for estimating matrices P and Q. One method consists of minimizing the squared error function with the observed entries of R:

$$e_{ij}^{2}={\left(r_{ij}-\sum_{k=1}^{K}p_{ik}q_{kj}\right)}^{2}+\frac{\lambda }{2}\sum_{k=1}^{K}\left({\left\|P\right\|}^{2}+{\left\|Q\right\|}^{2}\right),$$ (4)

where λ is the regularization parameter that controls overfitting. To minimize the squared error function, we have to know in which direction we have to modify the values of $p_{ik}$ and $q_{kj}.$ In other words, we need to know the gradient at the current values, and therefore we differentiated the above equation with respect to these two variables separately:

$$\frac{\partial }{\partial p_{ik}}e_{ij}^{2}=-2\left(r_{ij}-{\hat{r}}_{ij}\right)q_{kj}+\lambda p_{ik}=-\left(2e_{ij}q_{kj}-\lambda p_{ik}\right)$$

$$\frac{\partial }{\partial q_{kj}}e_{ij}^{2}=-2\left(r_{ij}-{\hat{r}}_{ij}\right)p_{ik}+\lambda q_{kj}=-\left(2e_{ij}p_{ik}-\lambda q_{kj}\right).$$

Since we have the gradient, next we provide the updating rules for both $p_{ik}$ and $q_{kj}$:

$$p_{ik}=p_{ik}+\alpha \frac{\partial }{\partial p_{ik}}e_{ij}^{2}=p_{ik}+\alpha \left(2e_{ij}q_{kj}-\lambda p_{ik}\right),$$ (5)

$$q_{kj}=q_{kj}+\alpha \frac{\partial }{\partial q_{kj}}e_{ij}^{2}=q_{kj}+\alpha \left(2e_{ij}p_{ik}-\lambda q_{kj}\right),$$ (6)

where $e_{ij}=r_{ij}-p_{ik}q_{kj}$ and α is the learning rate; we usually choose a small value, for example, 0.0002. The learning rate affects the learning time, and too large a value may lead to divergence. In general, a smaller learning rate gives better performance, but the learning time is also longer. Using the updated rules, we can iteratively perform the operation until the error converges to its minimum. Before implementing this gradient descendent algorithm, we need to select a learning rate α, a regularization coefficient λ, and the required number of latent features K. Also, we need to set the starting values of matrices P and Q. The R code for implementing this algorithm is provided in Appendix A2.

### Multiple-environment (ME) mixed model

For each trait, the following univariate linear mixed model is proposed:

$$y_{ij}=E_{i}+g_{j}+gE_{ij}+e_{ij},$$ (7)

where $y_{ij}$ represents the normal response from the jth line in the ith environment (i=1,2,…,I, j=1,2,…,J). For illustration purposes, we will use I=3. $E_{i}\;$represents the fixed effect of the ith environment and it is assumed as a fixed effect, $g_{j}$ represents the random effect of the genomic effect of the jth line, with $g={\left(g_{j},\ldots ,g_{J}\right)}^{T}∼N\left(0,\;G_{g}\right),$ and $G_{g}$ is of order J×J and represents the genomic relationship matrix. It was calculated using the VanRaden (2008) method as $G_{g}=WW^{T}/p,$ with **W** as the matrix of markers of order **J×p.** $gE_{ij}$ is the random interaction term between the genomic effect of the jth line and the ith environment, where $\mathrm{gE}={\left(gE_{11},\ldots ,gE_{IJ}\right)}^{T}∼N\left(0,\;I_{I}⊗G\right)$ and $e_{ij}$ is a random error term associated with the jth line in the ith environment distributed as $N\left(0,\;\sigma^{2}\right).$ As previously mentioned, this model was used for each of the l=1,…,L traits, where L denotes the number of traits under study.

### Multiple-trait and multiple-environment unstructured mixed model

To account for the correlation between traits, we stacked the information of all the *L* traits given in Equation (7). In matrix notation, the whole mixed model is equal to

$$Y=\mathrm{X\beta}+Z_{1}b_{1}+Z_{2}b_{2}+e\text{,}$$ (8)

where Y is of order **Ln×1,** X is of order **Ln×IL,** β is of order **IL×1** and contains the β coefficients of the environment–trait combinations, $Z_{1}$ is of order Ln×LJ, $b_{1}$ is of order LJ×1, $Z_{2}$ is of order Ln×IJL, $b_{2}$ is of order IJL×1, and e is of order **Ln×1.** Then $b_{1}∼N\left(0,\;G_{1}\right),$ $b_{2}∼N\left(0,\;G_{2}\right)$, and e∼N(0, R), where $G_{1}=G_{g}⊗\Sigma_{t},$ $\Sigma_{t}$ is the genetic covariance matrix between traits and is assumed unstructured, and ⊗ denotes a Kronecker product, $G_{2}=\Sigma_{E}⊗G_{1},$ where $\Sigma_{E}$ is assumed as a general matrix of order I×I. It is important to point out that the trait × environment (T × E) interaction term is included in the fixed effect β, while the trait × genotype (T × G) interaction term is included in the random effect $b_{1}$, and the three-way (T × G × E) interaction term is included in $b_{2}.$ The errors are assumed to be correlated with the covariance defined as $R=I_{n}⊗R_{e},$ where $R_{e}$ is the residual general covariance matrix between traits.

### Proposed methods

In this section, we present the proposed methods for analyzing MTME data with continuous phenotypes.

#### Method IBCF:

In this method, the prediction analysis is performed with IBCF using Equation (1) directly. However, the training of the multiple-trait and multiple-envirionmnet (MTME) data first need to be scaled for each trait–environment combination given in Table 3. When the genotypes under study are the same in all environments, we create a rectangular rating matrix where the number of rows is equal to the number of genotypes and the number of columns is equal to the resulting combinations of environments and traits; that is, if the number of traits is 4 and the number of environments is 5, then the resulting number of columns in this rating matrix should be 20 (Table 3). However, when the data are only multiple trait (MT) or ME, the rows should be genotypes, while the number of columns should be the phenotypes measured in each environment or trait. Table 3 gives an example of how the phenotypic information resulting from a MTME data set should be arranged to implement IBCF.

**Table 3:** Phenotypic information for building the rating matrix for multiple-trait and multiple-environment data for J genotypes, I environments, and L traits

	Environment–Trait Combinations									
Genotypes	E1T1	…	E1TL	E2T1	…	E2TL	…	EIT1	…	EITL
G1	$y_{111}$	…	$y_{11L}$	$y_{211}$	…	$y_{21L}$	…	$y_{I11}$	…	$y_{I1L}$
G2	$y_{121}$	…	$y_{12L}$	$y_{221}$	…	$y_{22L}$	…	$y_{I21}$	…	$y_{I2L}$
…	…	…	…	…	…	…	…	…	…	…
GJ	$y_{1J1}$	…	$y_{1JL}$	$y_{2J1}$	…	$y_{2JL}$	…	$y_{IJ1}$	…	$y_{IJL}$

G denotes genotypes, E denotes environment, T represents trait, and $y_{ijl}$ is the phenotype from the jth line in the ith environment for the lth trait.

#### Method MF:

This method implements the matrix factorization method described in Equations (2)–(6). To be correctly implemented, the data need to be placed as shown in Table 3; implementation was performed using the R code given in Appendix A2.

#### Method ME mixed model:

Method ME consists of using Equation (7) for each trait; it is thus a typical genomic-based model with the main effects of environments, genotypes, and the genotype × environment interaction term.

#### Method MTME mixed model:

Method MTME consists of using the following multitrait and multienvironment model:

$$y_{ijl}=E_{i}+g_{j}+T_{l}+gE_{ij}+TE_{il}+gT_{jl}+gET_{ijl}+e_{ijl},$$ (9)

where $y_{ijl}$ represents the normal response of the jth line in the ith environment for trait l (i=1,2,…,I, j=1,2,…,J, l=1,…,L). $T_{l}\;$ represents the fixed effect of the lth trait, $TE_{il}$ is the fixed interaction term between the lth trait and the ith environment, $gT_{jl}$ represents the random effect of the interaction of genotype j and the lth trait, with $\mathrm{gT}={\left(gT_{11},\ldots ,gT_{JL}\right)}^{T}∼N\left(0,G⊗I_{L}\right),$ $gET_{ijl}$ is the three-way interaction of genotype j,  the ith environment and the lth trait, with $\mathrm{gET}={\left(gET_{111},\ldots ,gET_{IJL}\right)}^{T}∼N\left(0,I_{I}⊗G⊗I_{L}\right),$ and $e_{ijl}$ is a random error term associated with the jth line, the *i*th environment and the *l*th trait is distributed as $N\left(0,\;\sigma^{2}\right).$

### Simulated data sets

For testing the proposed models and methods, we simulated MTME data using Equation (8) with three environments, three traits, and 1000 genotypes, along with one replication for environment-trait-genotype combinations. We assumed that $\beta^{T}=[$15,8,7,12,6,7,14,9,8], where the first three beta coefficients belong to traits 1, 2, and 3 in environment 1, the next three values to the three traits in environment 2, and the last three to environment 3. Also, this first set of variance-covariance matrices were assumed to be $\Sigma_{t}=\left[\begin{array}{ccc} 0.900 & 0.721 & 0.765\\ 0.721 & 0.800 & 0.721\\ 0.765 & 0.721 & 0.900 \end{array}\right],$ $\Sigma_{E}=\left[\begin{array}{ccc} 0.500 & 0.4846 & 0.5205\\ 0.4846 & 0.6500 & 0.5935\\ 0.5205 & 0.5934 & 0.7500 \end{array}\right]$, and $R_{e}=\left[\begin{array}{ccc} 0.450 & 0.3695 & 0.3275\\ 0.3695 & 0.4200 & 0.3164\\ 0.3276 & 0.3164 & 0.330 \end{array}\right]$. These three variance-covariance matrices gave rise to a correlation of 0.85 between each pair of traits (genetic and residual) and between each pair of environments. We also assumed that the genomic relationship matrix is known, $G_{g}=0.7I_{1000}+0.3J_{1000},$ where $I_{1000}$ is an identity matrix of order 1000 and $J_{1000}$ is a matrix of order 1000×1000  of ones. Therefore, the total number of observations was 3×1000×3×1=9000, that is, 3000 for each trait. With these parameters, we simulated three data sets which were used for testing the prediction accuracy of the proposed models under each of the three studied scenarios. These scenarios included (S1), which generated the data as normal data using Equation (8); (S2), where the data were also generated with Equation (8) but with the error term replaced by exp[−1.25 × abs(e)], where e is exactly the same as what was generated under the first scenario, however, it now produced negative skewed data; and (S3), the last scenario, which also generated the data with Equation (8) but with the error term replaced by exp(1.25 × abs(e)) to induce positive skewed data.

Additionally, under the same conditions as mentioned above, we studied two other sets of variance-covariance matrices: set 2, with $\Sigma_{t}=\left[\begin{array}{ccc} 0.900 & 0.424 & 0.45\\ 0.424 & 0.800 & 0.424\\ 0.45 & 0.424 & 0.900 \end{array}\right],$ $\Sigma_{E}=\left[\begin{array}{ccc} 0.500 & 0.285 & 0.3061\\ 0.285 & 0.6500 & 0.349\\ 0.306 & 0.349 & 0.7500 \end{array}\right]$, and $R_{e}=\left[\begin{array}{ccc} 0.450 & 0.217 & 0.193\\ 0.217 & 0.4200 & 0.186\\ 0.193 & 0.186 & 0.330 \end{array}\right]$; and set 3, with $\Sigma_{t}=\left[\begin{array}{ccc} 0.900 & 0.212 & 0.225\\ 0.212 & 0.800 & 0.212\\ 0.225 & 0.212 & 0.900 \end{array}\right],$ $\Sigma_{E}=\left[\begin{array}{ccc} 0.500 & 0.1425 & 0.1531\\ 0.1425 & 0.6500 & 0.1745\\ 0.1531 & 0.1745 & 0.7500 \end{array}\right]$, and $R_{e}=\left[\begin{array}{ccc} 0.450 & 0.1086 & 0.0963\\ 0.1086 & 0.4200 & 0.0931\\ 0.0963 & 0.0931 & 0.330 \end{array}\right]$. The first set of variance-covariance matrices produced a pair of correlations between traits and between environments of 0.85. The second set produced a pair of correlations between traits and between environments of 0.5, while the third set of variance-covariance matrices produced a pair of correlations between traits and between environments of 0.25. These three sets of variance-covariance matrices were proposed for studying the performance of the methods proposed in the context of high correlation (first set of variance-covariances), medium correlation (second set of variance-covariances), and low correlation (third set of variance-covariances) between traits (genetic and residual) and between environments.

### Experimental data sets

Here we present information on the data sets used for implementing the proposed methods. In total, three data sets were used (one of maize and two of wheat).

#### Data set 1:

A total of 250 wheat lines were extracted from a large set of 39 yield trials grown during the 2013–2014 crop season in Ciudad Obregon, Sonora, Mexico (Rutkoski *et al.* 2016). The trials were sown in mid-November and grown on beds with five and two irrigations, in addition to drip irrigation. Days to heading (HD) was recorded as the number of days from germination until 50% of spikes had emerged in each plot in the first replicate of each trial. Grain yield (GY) was the total plot grain yield measured after maturity, and plant height (PH) was recorded in centimeters.

Image data of the yield trials were collected using a hyperspectral camera (A-series, Mirco-Hyperspec VNIR; Headwall Photonics, Fitchburg, MA) mounted on a manned aircraft, which allowed us to calculate vegetative indices for each plot. The green normalized difference vegetation index (GNDVI) was one of the traits used in this study, since it is considered a good predictor when used with pedigree and/or genomic prediction of GY in wheat due to its high heritability and genetic correlation. Trait GNDVI can also be measured remotely on large numbers of candidates for selection.

Genotyping-by-sequencing (GBS) was used for genome-wide genotyping. Single nucleotide polymorphisms (SNPs) were called across all lines using the TASSEL GBS pipeline anchored to the genome assembly of Chinese Spring. SNP calls were extracted, and markers were filtered so that the percent of missing data did not exceed 80%. Individuals with >80% missing marker data were removed, and markers were recorded as −1, 0, and 1, indicating homozygous for the minor allele, heterozygous, and homozygous for the major allele, respectively. Next, markers with <0.01 minor allele frequency were removed, and missing data were imputed with the marker mean. A total of 12,083 markers remained after marker editing.

#### Data set 2:

A total of 309 doubled haploid maize lines were phenotyped and genotyped; they are part of the data set used by Crossa *et al.* (2013) and Montesinos-López *et al.* (2016), which is comprised of a total of 504 doubled haploid lines derived by crossing and backcrossing eight inbred lines to form several full-sib families. Traits available in this data set include grain yield (GY), anthesis-silking interval (ASI), and plant height (PH); each of these traits was evaluated in three optimum rain-fed environments (Env1, Env2, and Env3). The experimental field design in each of the three environments was an α-lattice incomplete block design with two replicates. Data were preadjusted using block estimates, while environmental effects were derived from a linear model that accounted for the incomplete block design within environment and for environmental effects.

The genomic data were obtained with GBS for each maize chromosome. The number of markers after initial filtering and the number of markers after imputation were summarized in Crossa *et al.* (2013). Filtering was first done by removing markers that had >80% of the maize lines with missing values; then markers with a minor allele frequency ≤0.05 were deleted. The total number of GBS data were 681,257 SNPs and, after filtering for missing values and minor allele frequency, 158,281 SNPs were used for the analyses. About 20% of cells were missing in the filtered GBS information used for prediction; these missing values were replaced by their expected values before doing the prediction.

#### Data set 3:

This third data set was composed of 26,264 wheat lines that were planted over 3 yr (year_13_14; year_14_15, and year_15_16). In the first year (year_13_14), 7672 lines were planted, in the second year (year_14_15) 9091 lines were planted, and in the third year (year_15_16) the remaining 9501 lines were planted. The following five traits were measured on each line: days to heading (HD), days to maturity (DMT), plant height (PH), lodging, and grain yield (GY).

### Assessing prediction accuracy

To assess prediction accuracy, 20 training–testing random partitions were implemented, as well as two types of cross-validations. The first one (CV1) mimicked a situation where lines were evaluated in some environments for the traits of interest; however, some lines were missing in all the other environments. In this cross-validation, we assigned 80% of the lines to the training set and the remaining 20% to the testing set. The second cross-validation (CV2) mimicked a situation where we wanted to predict all the information of one trait for a complete year; however, the available information was for a previous year for all the traits under study and, for the target year, we had information for several traits except the one of interest. We used the Pearson correlation to compare the predictive phenotipic values to the observed phenotype. Models with higher correlation values had better predictions.

In the above-mentioned case, cross-validation CV1 was implemented for the simulated data sets and the first two real data sets (Data set 1 and Data set 2), while cross-validation CV2 was implemented for the real Data set 3. It is important to point out that in Data set 3, there were no common lines across years. Also, to illustrate how to predict one trait for the whole year, we assumed that one trait was missing. For example, we assumed that trait GY was missing in the year_14_15 (9091 lines missing for GY) and the information on the training set was the information from year_13_14 and year_14_15, but with GY missing in year_14_15. To predict GY for year_15_16, we used the information from year_13_14, year_14_15 and year_15_16 as the training set, but with GY missing in year_15_16 (9501 records missing for GY in year_15_16). The same was done when another trait was assumed to be missing.

### Data availability

The maize and wheat phenotypic and genotypic data sets used in this study can be downloaded from the link: http://hdl.handle.net/11529/11099. The maize phenotypic and genotypic data sets are Maize_data.RData and Gg.RData, respectively. The wheat phenotypic and genotypic data sets are Data.Trigo.RData and G.Trigo.RData, respectively. The wheat large data set is Large_wheat_data.RData.

## Results

The results are organized into two main sections. The first section presents the results in terms of prediction accuracy for the simulated data, while the second section gives the prediction accuracy of the four proposed methods but for the real data sets.

### Prediction accuracies using simulated data

**Figure 1:**
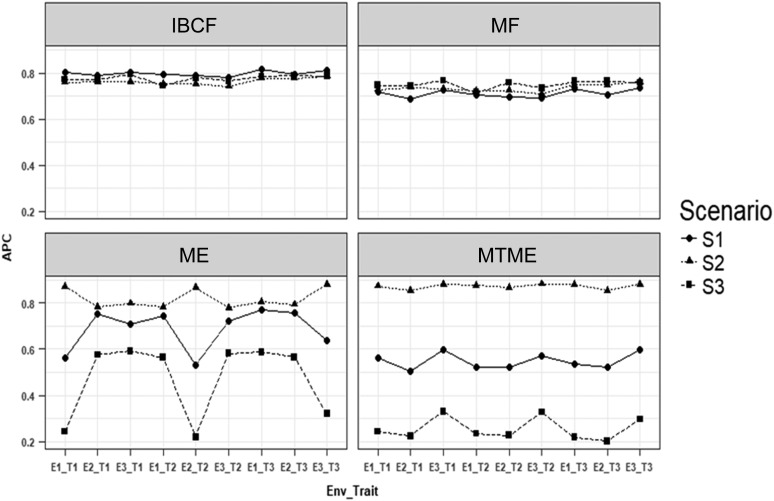
Simulated data. Average Pearson correlation (APC) for each environment–trait combination using the four methods under study [IBCF, matrix factorization (MF), ME, and MTME] for data simulated with a correlation of traits (genetic and residual) and correlation of environments of 0.85. S1 is the scenario under normality, S2 is the scenario under the error negative skew multiplied by 1.25, and S3 represents the scenario under the error positive skew multiplied by 1.25. The notation E1_T1 means environment 1, trait 1.

**Figure 2:**
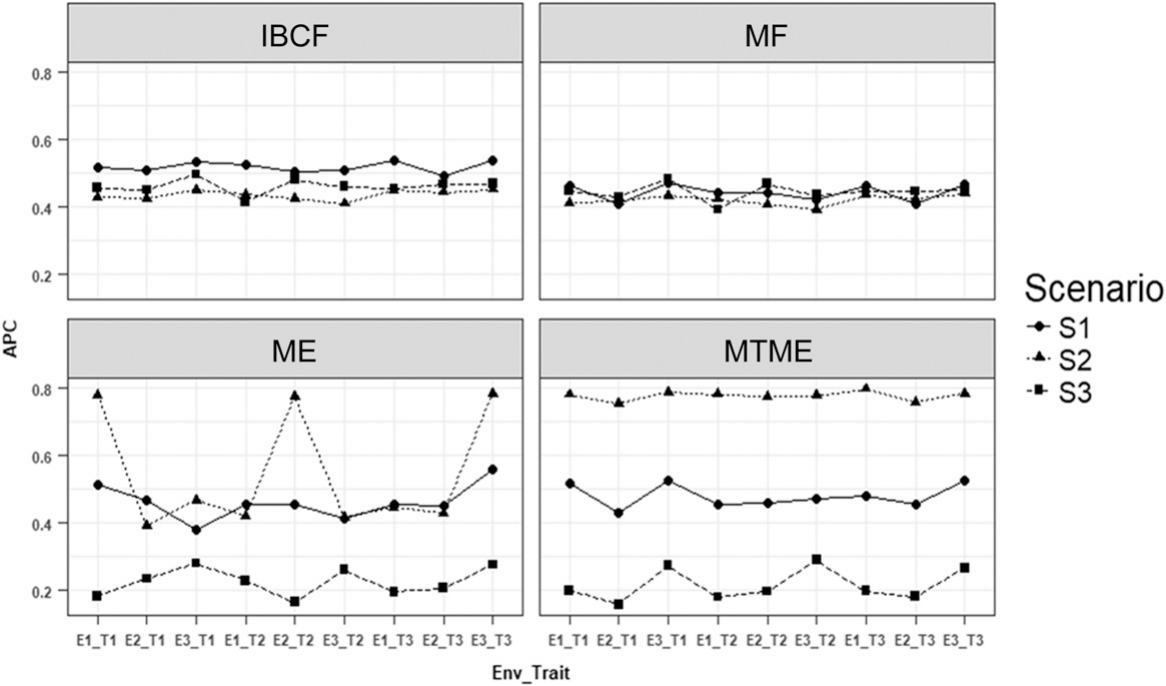
Simulated data. Average Pearson correlation (APC) for each environment-trait combination using the four methods being studied [IBCF, matrix factorization (MF), ME, and MTME] for data simulated with a correlation of traits (genetic and residual) and correlation of environments of 0.5. S1 is the scenario under normality, S2 is the scenario under the error negative skew multiplied by 1.25, and S3 represents the scenario under the error positive skew multiplied by 1.25. The notation E1_T1 means environment 1, trait 1.

**Figure 3:**
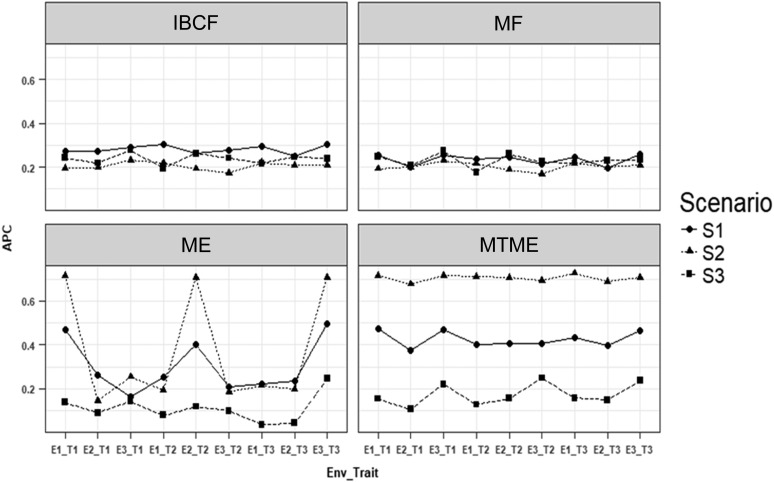
Simulated data. Average Pearson correlation (APC) for each environment-trait combination using the four methods under study [IBCF, matrix factorization (MF), ME, and MTME] for data simulated with a correlation of traits (genetic and residual) and correlation of environments of 0.25. S1 is the scenario under normality, S2 denotes the scenario under the error negative skew multiplied by 1.25, and S3 is the scenario under the error positive skew multiplied by 1.25. The notation E1_T1 means environment 1, trait 1.

What follows are the prediction accuracies for each of the three studied scenarios obtained using the four proposed methods. Figure 1 shows the results of using a correlation between traits (genetic and residual) and between environments of 0.85 (high correlation), while Figure 2 depicts the results when the correlation assumed between traits (genetic and residual) and between environments was 0.5 (intermediate correlation). Finally, Figure 3 displays the results when the correlation assumed between traits (genetic and residual) and between environments was 0.25 (low correlation).

Figure 1 shows that under the first scenario (when the data were normal), method IBCF was the best in terms of prediction accuracy for all the trait-environment combinations. The second-best method was matrix factorization and the worst was method MTME. It is important to point out that, on average, method IBCF was 10.8% better than matrix factorization, 13.8% better than method ME, and 31.3% better than MTME. Under scenario S2 (considering the residual negative skew multiplied by 1.25), we observed that method MTME was the best, followed by method ME, then by IBCF, and finally matrix factorization, which was the worst. However, the gains in terms of average predictions of method MTME with regard to methods IBCF, matrix factorization, and ME were only 12.5, 15.8, and 6.2%, respectively (Figure 1). Under scenario S3 (with the residual positive skew multiplied by 1.25), method IBCF was the best, followed by matrix factorization; MTME was the worst and, on average, IBCF was better than methods matrix factorization, ME, and MTME by 3.3, 39.3, and 67.1%, respectively (Figure 1). Appendix A7 gives the SE of the average Pearson correlations for each scenario and environment–trait combination of the predictions given in Figure 1, Figure 2, and Figure 3.

Figure 2 shows that in the first scenario (when the data were normal), method IBCF (collaborative filtering) was the best in terms of prediction accuracy, as shown in seven out of nine trait–environment combinations, as opposed to matrix factorization, which was the worst. Also, it must be noted that, on average, method IBCF was 14.5% better than matrix factorization, 11.3% better than ME, and 7.7% better than MTME. Under scenario S2 (with the residual negative skew multiplied by 1.25), method MTME was the best, while matrix factorization was the worst; MTME was, on average, 43.8% better than IBCF, ∼45.7% better than matrix factorization, and 29.9% better than ME (Figure 2). Under scenario S3 (with the residual positive skew multiplied by 1.25), method IBCF was the best, MTME was the worst and, on average, IBCF was better than methods matrix factorization, ME, and MTME by 3.62, 51.3, and 53.3%, respectively (Figure 2).

Figure 3 shows that under the first scenario (when the data were normal), method MTME was the best in terms of prediction accuracy, as shown by the results of all the environment–trait combinations; the second best was ME, and the worst was matrix factorization. On average, MTME was 34% better than IBCF, 44.9% better than matrix factorization, and 29.1% better than ME. Under scenario S2 (with the residual negative skew multiplied by 1.25), method MTME was the best, matrix factorization was the worst, and MTME was, on average, better than IBCF, matrix factorization, and ME by 70.9, 71.4, and 47.7%, respectively (Figure 3). Under scenario S3 (with the residual positive skew multiplied by 1.25), method IBCF was the best, followed by matrix factorization; ME was the worst and, on average, IBCF was better than methods matrix factorization, ME, and MTME by 3.0, 53.4, and 27.1%, respectively (Figure 3). In general, method IBCF was poorer when the correlation between traits (genetic and residual) and between environments was low; however, in Figure 3 under scenario (S3), we can see than sometimes it was better.

### Prediction accuracy using real data sets

In this section, we present the results of predictions under the four proposed methods for the three real data sets. We begin by presenting the results obtained using the first data set (wheat data set), followed by the results for the second data set (maize data set), and finally, the results for the third real data set (large wheat data set).

In Table 4 we present the prediction accuracies using the wheat data set under method MF with 10 different values of the regularization parameter lambda (λ). The table shows that the worst predictions were made without the regularization parameter, that is, when λ=0. Then it shows that when *λ* = 0.4, the prediction accuracy increased with regard to λ=0 by 15.5%, continuing to increase the value of *λ* until 1.6, the increase in the average prediction accuracy is up to 0.594 (21.88%). However, after a value of *λ* = 2.2, the prediction accuracy starts to decrease. This implies that to get the best performance of method matrix factorization, various values of the parameter *λ* need to be tested before doing the final implementation of this model.

**Table 4:** Wheat data set 1

	λ=0		λ=0.4		λ=0.8		λ=1.2		λ=1.4	
Env–Trait	Mean	SE	Mean	SE	Mean	SE	Mean	SE	Mean	SE
Bed2IR_HD	0.773	0.022	0.817	0.012	0.835	0.009	0.858	0.010	0.858	0.010
Bed2IR_GNDVI	0.627	0.014	0.659	0.013	0.660	0.011	0.662	0.011	0.661	0.011
Bed2IR_GY	0.496	0.027	0.523	0.016	0.513	0.014	0.502	0.014	0.499	0.014
Bed2IR_PH	0.479	0.033	0.530	0.019	0.616	0.012	0.626	0.010	0.621	0.010
Bed5IR_HD	0.765	0.029	0.811	0.012	0.854	0.007	0.873	0.007	0.876	0.007
Bed5IR_GNDVI	0.453	0.042	0.529	0.016	0.548	0.015	0.560	0.013	0.563	0.013
Bed5IR_GY	−0.018	0.027	0.078	0.027	0.077	0.030	0.078	0.030	0.077	0.031
Bed5IR_PH	0.058	0.053	0.264	0.031	0.367	0.020	0.382	0.022	0.383	0.023
Drip_HD	0.872	0.011	0.872	0.009	0.900	0.005	0.906	0.004	0.908	0.004
Drip_GNDVI	0.546	0.025	0.537	0.021	0.554	0.018	0.560	0.018	0.562	0.018
Drip_GY	0.209	0.076	0.466	0.024	0.472	0.020	0.462	0.020	0.461	0.020
Drip_PH	0.301	0.036	0.492	0.025	0.589	0.022	0.636	0.018	0.652	0.017
Average	0.464	0.033	0.548	0.019	0.582	0.015	0.592	0.015	0.593	0.015
	λ=1.6		λ=1.8		λ=2.2		λ=2.6		λ=3	
Bed2IR_HD	**0.864**	0.011	0.864	0.011	0.859	0.012	0.856	0.012	0.856	0.011
Bed2IR_GNDVI	**0.660**	0.012	0.657	0.012	0.646	0.012	0.642	0.013	0.641	0.013
Bed2IR_GY	**0.495**	0.014	0.491	0.015	0.480	0.015	0.465	0.016	0.459	0.016
Bed2IR_PH	**0.617**	0.011	0.612	0.011	0.604	0.012	0.601	0.013	0.597	0.014
Bed5IR_HD	**0.877**	0.007	0.877	0.007	0.874	0.007	0.871	0.007	0.869	0.007
Bed5IR_GNDVI	**0.565**	0.012	0.567	0.012	0.571	0.012	0.572	0.012	0.574	0.012
Bed5IR_GY	**0.077**	0.031	0.076	0.031	0.075	0.031	0.076	0.030	0.078	0.030
Bed5IR_PH	**0.383**	0.023	0.384	0.023	0.385	0.023	0.382	0.022	0.381	0.022
Drip_HD	**0.909**	0.004	0.909	0.003	0.908	0.003	0.906	0.003	0.906	0.003
Drip_GNDVI	**0.563**	0.017	0.564	0.017	0.563	0.017	0.563	0.017	0.565	0.017
Drip_GY	**0.462**	0.020	0.462	0.020	0.463	0.021	0.460	0.021	0.453	0.022
Drip_PH	**0.660**	0.016	0.664	0.016	0.667	0.016	0.670	0.015	0.670	0.015
Average	**0.594**	0.015	0.594	0.015	0.591	0.015	0.589	0.015	0.587	0.015

Prediction accuracies with Pearson correlation for each environment–trait (Env–Trait) combination of the matrix factorization model (MF) with different values of lambda (λ) under cross-validation scheme CV1. The best predictions of the four methods are in boldface, and the comparisons are made by row. Wheat data set obtained from Rutkoski *et al.* (2016**)**.

In Table 5, we compare the predictions of the four proposed methods using the first data set (wheat data set). Table 5 indicates that IBCF without markers was the best method in terms of prediction accuracy, as shown by six out of the 12 environment-trait combinations; the second best was method matrix factorization, and the worst was method ME with and without markers. Method IBCF without markers was, on average, better than methods IBCF with markers, matrix factorization and ME without markers, ME with markers, MTME without markers, and MTME with markers by 44.6, 1.7, 99.2, 99.5, 29.9, and 36.3%, respectively. These findings of the good performance of method IBCF are related to the phenotypic correlations between traits, which are not low, as can be seen in Appendix A4. Here it is important to point out that to take into account the marker information, the genomic relationship matrix created with the marker information was used as a proxy for the phenotypic correlation between genotypes (users). Then we applied the UBCF instead of the IBCF. However, according to these results, there is evidence that using the genomic relationship matrix alone as a proxy for the phenotypic correlation between genotypes is not very reasonable.

**Table 5:** Wheat data set 1

	IBCF				MF		ME				MTME			
	No Markers		With Markers		No Markers		No Markers		With Markers		No Markers		With Markers	
Env–Trait	Mean	SE	Mean	SE	Mean	SE	Mean	SE	Mean	SE	Mean	SE	Mean	SE
Bed2IR_HD	0.875	0.011	0.374	0.023	0.864	0.011	−0.005	0.030	−0.022	0.030	0.829	0.008	**0.880**	0.006
Bed2IR_GNDVI	**0.664**	0.012	0.411	0.020	0.660	0.012	−0.012	0.023	−0.012	0.023	0.154	0.027	0.040	0.022
Bed2IR_GY	**0.557**	0.012	0.353	0.018	0.495	0.014	−0.014	0.024	0.043	0.022	0.181	0.024	0.098	0.025
Bed2IR_PH	**0.647**	0.011	0.325	0.023	0.617	0.011	−0.022	0.016	0.026	0.015	0.235	0.014	0.031	0.015
Bed5IR_HD	0.873	0.007	0.390	0.015	0.877	0.007	−0.006	0.028	0.024	0.028	0.866	0.01	**0.893**	0.008
Bed5IR_GNDVI	**0.586**	0.011	0.249	0.022	0.565	0.012	−0.008	0.019	0.005	0.019	0.001	0.033	−0.003	0.033
Bed5IR_GY	0.091	0.032	0.035	0.021	0.077	0.031	0.019	0.024	0.024	0.024	**0.403**	0.022	0.393	0.027
Bed5IR_PH	0.410	0.022	0.345	0.018	0.383	0.023	0.063	0.021	−0.013	0.025	**0.603**	0.012	0.505	0.015
Drip_HD	0.920	0.003	0.464	0.023	0.909	0.004	0.018	0.023	0.006	0.023	0.917	0.004	**0.922**	0.003
Drip_GNDVI	**0.568**	0.016	0.371	0.031	0.563	0.017	0.047	0.034	−0.005	0.036	−0.12	0.027	−0.043	0.026
Drip_GY	0.401	0.024	0.362	0.018	**0.462**	0.020	−0.025	0.027	−0.015	0.027	0.432	0.019	0.364	0.017
Drip_PH	**0.657**	0.016	0.335	0.022	0.660	0.016	0.003	0.017	−0.021	0.017	0.579	0.022	0.538	0.022
Average	**0.604**	0.015	0.335	0.021	0.594	0.015	0.005	0.024	0.003	0.024	0.424	0.018	0.385	0.018

Prediction accuracies with Pearson correlation for each environment–trait (Env–Trait) combination of the proposed methods for the wheat data set from Rutkoski *et al.* (2016**)**, under cross-validation scheme CV1. The best predictions of the four methods are in boldface, and the comparisons are made by row. “No markers” means that genomic information was not used, while “with markers” means that genomic information was used. MF, matrix factorization.

Table 6 indicates that for the second data set (maize data set), the best prediction accuracies were observed under ME with markers (univariate model), followed by model MTME with markers (multivariate model), and the worst were observed under model matrix factorization without makers. In general, the predictions of ME were slightly better than those of MTME with and without markers; the second best were obtained using method IBCF with markers, while the worst were obtained using matrix factorization without markers. Methods ME and MTME were considerably better than the remaining methods. ME and MTME with markers were ∼30.9% better than method IBCF without markers, 27.8% better than IBCF with markers, 35.4% better than matrix factorization without markers, 37.5% better than ME without markers, and 37.7% better than MTME without markers. Here it is important to point out that when methods IBCF, ME, and MTME took into account the genomic relationship matrix, the predictions improved. However, the improvement of IBCF by taking into account the markers with regard to IBCF without markers was only 4.4%. In general, the predictions with this real data set were low, which is mainly explained by the low phenotypic correlations between the nine environment–trait combinations, as can be seen in Appendix A5.

**Table 6:** Maize data set 2

	IBCF				MF		ME				MTME			
	No Markers		With Markers		No Markers		No Markers		With Markers		No Markers		With Markers	
Env–Trait	Mean	SE	Mean	SE	Mean	SE	Mean	SE	Mean	SE	Mean	SE	Mean	SE
EBU_GY	0.232	0.017	0.214	0.023	0.209	0.013	0.236	0.019	0.345	0.018	0.233	0.020	**0.353**	0.019
EBU_ASI	0.375	0.020	0.434	0.012	0.379	0.019	0.310	0.018	**0.510**	0.015	0.326	0.019	0.495	0.014
EBU_PH	0.214	0.022	0.421	0.018	0.188	0.021	0.123	0.020	**0.515**	0.014	0.117	0.022	0.485	0.016
KAK_GY	0.281	0.023	0.285	0.022	0.269	0.026	0.269	0.017	**0.401**	0.021	0.263	0.019	0.399	0.023
KAK_ASI	0.332	0.016	0.272	0.018	0.336	0.018	0.298	0.020	**0.418**	0.018	0.316	0.019	0.412	0.017
KAK_PH	0.317	0.023	0.272	0.023	0.234	0.024	0.260	0.019	0.409	0.020	0.278	0.019	**0.452**	0.022
KTI_GY	0.206	0.016	0.197	0.018	0.190	0.018	0.236	0.015	0.299	0.018	0.234	0.018	**0.306**	0.020
KTI_ASI	0.269	0.020	0.083	0.024	0.286	0.019	0.303	0.017	**0.273**	0.022	0.264	0.015	0.239	0.021
KTI_PH	0.282	0.019	0.445	0.014	0.253	0.022	0.235	0.019	0.475	0.016	0.233	0.020	**0.491**	0.015
Average	0.279	0.019	0.292	0.019	0.261	0.020	0.252	0.018	0.405	0.018	0.252	0.019	0.404	0.019

Prediction accuracies with Pearson correlation for each environment–trait (Env–Trait) combination of the proposed methods for the maize data set under cross-validation scheme CV1. The best predictions of the seven methods are in boldface, and the comparisons are made by row. “No markers” means that genomic information was not used, while “with markers” means that genomic information was used. MF, matrix factorization.

Table 7 shows the results for the third real data set (the large wheat data set), where only methods IBCF and matrix factorization were implemented. Method matrix factorization was implemented with three values of latent features (K=2,3,4). It is important to recall that in this data set there are five columns (items) in the rating matrix (GY, HD, DMT, PH, and lodging) and 36,181 rows (observations), with each row corresponding to a different wheat line. The lines were evaluated in 4 yr: 7672 were evaluated in year_13_14, 9091 were evaluated in year_14_15, 9501 were evaluated in year_15_16, and 9917 were evaluated in year_16_17. In this cross-validation scheme (CV2), we assumed trait GY was missing in the following year and predicted using as training data 1, 2, or 3 yr before. For example, when GY was predicted in year_14_15, only the information of year_13_14 and year_14_15 was used as training data, with 9091 lines missing trait GY in year_14_15 to be predicted. When 9501 lines (missing trait GY) were predicted for year_15_16, two scenarios were studied: (1) when only one previous year was used as training and denoted as GY_Year_15_16_1yb; and (2) when two previous years were used as training and denoted as GY_Year_15_16_2yb. Finally, when 9917 lines (missing trait GY) were predicted for year_16_17, three scenarios were studied: (1) when only one previous year was used as training, denoted as GY_Year_16_17_1yb; (2) when two previous years were used as training, denoted as GY_Year_16_17_2yb; and (3) when three previous years, denoted as GY_Year_16_17_3yb, were used as training sets. When the other traits were predicted, the same scenarios were studied, and the corresponding trait to be predicted was assumed to be missing.

**Table 7:** Large wheat data set 3

	IBCF		MF, *K* = 2		MF, *K* = 3		MF, *K* = 4	
Env–Trait	Mean	PCL	Mean	PCL	Mean	PCL	Mean	PCL
GY_Year_14_15	0.333	33.30	0.311	33.30	**0.374**	33.3	0.353	33.3
GY_Year_15_16_1yb	**0.335**	33.80	0.254	33.80	0.241	33.8	0.240	33.8
GY_Year_15_16_2yb	**0.305**	33.75	0.234	33.75	0.198	33.75	0.197	33.75
GY_Year_16_17_1yb	**0.285**	28.30	−0.178	28.30	−0.178	28.30	−0.179	28.30
GY_Year_16_17_2yb	**0.286**	28.15	0.193	28.15	0.222	28.15	0.230	28.15
GY_Year_16_17_3yb	**0.287**	28.25	0.148	28.25	0.193	28.25	0.196	28.25
Average	**0.305**	30.93	0.160	30.93	0.175	30.93	0.17	30.93
HD_Year_14_15	**0.627**	53.65	0.483	53.65	0.556	53.65	0.565	53.65
HD_Year_15_16_1yb	0.508	48.35	0.320	48.35	**0.616**	48.35	0.607	48.35
HD_Year_15_16_2yb	0.537	49.45	0.440	49.45	**0.567**	49.45	0.563	49.45
HD_Year_16_17_1yb	**0.650**	47.55	0.335	47.55	0.564	47.55	0.571	47.55
HD_Year_16_17_2yb	**0.646**	47.70	0.554	47.70	0.602	47.7	0.611	47.7
HD_Year_16_17_3yb	0.637	47.20	0.589	47.20	0.655	47.2	**0.656**	47.2
Average	**0.601**	48.98	0.454	48.98	0.59	48.98	0.60	48.98
DMT_Year_14_15	**0.647**	49.50	0.427	49.50	0.546	49.50	0.566	49.50
DMT_Year_15_16_1yb	0.491	39.55	0.510	39.55	**0.697**	39.55	0.694	39.55
DMT_Year_15_16_2yb	**0.636**	48.85	0.460	48.85	0.596	48.85	0.625	48.85
DMT_Year_16_17_1yb	0.527	37.30	0.270	37.30	**0.582**	37.30	0.580	37.30
DMT_Year_16_17_2yb	0.566	40.35	0.494	40.35	**0.611**	40.35	**0.611**	40.35
DMT_Year_16_17_3yb	0.548	38.90	0.565	38.90	**0.592**	38.90	0.588	38.90
Average	0.569	42.41	0.454	42.41	**0.60**	42.41	**0.61**	42.41
PH_Year_14_15	0.015	20.30	**0.088**	20.00	0.044	20	−0.014	20
PH_Year_15_16_1yb	0.026	20.40	**0.069**	20.40	−0.010	20.40	0.057	20.4
PH_Year_15_16_2yb	0.044	21.35	**0.092**	21.35	0.051	21.35	0.058	21.35
PH_Year_16_17_1yb	0.217	27.60	**0.226**	27.60	0.225	27.6	0.187	27.6
PH_Year_16_17_2yb	**0.243**	29.25	0.229	29.25	0.228	29.25	0.197	29.25
PH_Year_16_17_3yb	0.238	28.95	0.166	28.95	**0.241**	28.95	0.223	28.95
Average	0.131	24.64	0.145	24.59	0.13	24.59	0.12	24.59
Lodging_Year_14_15	**0.347**	40.70	0.104	40.70	0.120	40.70	0.124	40.70
Lodging_Year_15_16_1yb	−0.195	11.95	0.024	31.45	0.060	31.45	**0.074**	31.45
Lodging_Year_15_16_2yb	−0.274	9.65	0.099	37.25	0.115	37.25	**0.140**	37.25
Average	−0.041	20.77	0.076	36.467	0.098	36.47	**0.11**	36.47

Prediction accuracies with Pearson correlation for all genotypes missing in the years: GY_Year_14_15, GY_Year_15_16, and GY_Year_16_17 for the wheat data set under cross-validation scheme CV2. Here, only the IBCF and MF methods were implemented. Method MF was implemented with three values of latent features (*K* = 2, 3, 4). Traits in 1 yr were predicted with data from 1 yr before (1yb), 2 yr before (2yb), and 3 yr before (3yb). PCL denotes the percentage of common lines in the top 2000 lines. MF, matrix factorization.

The results in Table 7 indicate that when we predicted GY with IBCF in all six scenarios, the average prediction in terms of the Pearson correlation was ∼0.305, with an average agreement of 30.93% among the first 2000 lines predicted and observed. Under matrix factorization, GY predictions were lower, with Pearson correlations between 0.16 (with *K* = 2 latent features) and 0.175 (with *K* = 3); however, it is interesting to point out that under the three latent features used, the average agreement among the first 2000 lines predicted and observed was also 30.93%. When the HD trait was predicted under all six scenarios, good performance was observed, as the average prediction for the Pearson correlation was 0.601 under IBCF, 0.454 under matrix factorization with *K* = 2, 0.59 under matrix factorization with *K* = 3, and 0.60 under matrix factorization with *K* = 4, with average agreement of 48.33% in the first 2000 lines predicted and observed using the two methods. Similar behavior was observed when predicting the DMT trait, as the average prediction accuracy was 0.569 (Pearson correlation) under IBCF, 0.454 under matrix factorization with *K* = 2, 0.60 with *K* = 3, and 0.61 under matrix factorization with *K* = 4. In this trait, an average agreement of 41.6% in the first 2000 lines was predicted and observed for the two methods. On the other hand, when we predicted the traits PH and lodging, the average predictions were really poor: lower than 0.1145 for PH using the two methods and lower than 0.11 for lodging using the two methods. Additionally, the average agreements in the first 2000 lines predicted and observed were 24.642 and 20.77% for traits PH and lodging, respectively. The results in Table 6 show that methods IBCF and matrix factorization did a good job of predicting HD and DMT, and a reasonable job of predicting GY. However, they did a poor job of predicting PH and lodging. These contrasting results are due to the level of phenotypic correlation (see Appendix A6) since the traits that had high correlation with at least one other trait showed higher prediction accuracy and vice versa; that is, traits with low correlation with other traits were found to have low prediction accuracies. Also in Table 7 it is clear that for this data set, model IBCF was better than model matrix factorization.

## Discussion

In this article, we propose using two methods for recommender systems: IBCF and matrix factorization for predicting multiple traits of some genotypes that are missing in some environments. Our results using simulated and real data sets are interesting, since we found that when the phenotypic correlation between traits and environments is reasonably high, we can make predictions with reasonable accuracy (as shown in the simulation study presented) with both methods IBCF and matrix factorization, but method IBCF was the best. This is a very good sign, since the implementation of method IBCF is straightforward, as it is only necessary to place the information in a rectangular rating matrix where the rows are the genotypes and the columns represent the trait–environment combinations. Then, we need to scale each column by subtracting its mean and dividing by its SD. Obviously, this scaling process should be done only on the training data. Then, using the R code provided in Appendix A1, we can apply the IBCF technique. See the example in Appendix A3 to understand how to implement this algorithm.

We also provided the R code (Appendix A2) for implementing the matrix factorization method. However, the implementation of matrix factorization, even though it produced competitive predictions, was lower than that produced by method IBCF (using the R code given in Appendix A1). For its successful application, the following should to be taken into account: (1) first obtain the tuning parameter *λ* using cross-validation, (2) choose the number of latent features (parameter K) also using cross-validation; and (3) obtain appropriate starting values to get convergence. Since the IBCF is extremely fast, we suggest using these values as starting values for implementing the matrix factorization method. See the example in Appendix A3 to learn how to implement the matrix factorization algorithm.

From our results with simulated and real data sets, we found that the IBCF technique (method IBCF) is very competitive even compared to the MTME model for positive skew data. Under most scenarios where there was moderately high phenotypic correlation, the IBCF technique outperformed (in terms of prediction accuracy and implementation time) the MTME. The IBCF technique is very efficient in terms of the computational time required for its implementation and can be applied on large data sets. These techniques (IBCF and matrix factorization) can also be applied to MT or ME analysis only, as was done on the third real data set (large wheat data set). However, the placement of the data are slightly different, since in the rectangular rating matrix the rows are the genotypes but the columns are the phenotypes of traits or the phenotypes of environments. The expected predictions depend on the degree of the phenotypic correlation between traits or environments. We believe that these approaches could be very useful when the number of traits or environments, as well as the correlations between them, are large. The results obtained for the large wheat data set are very promising mostly under method IBCF, since they show that when the phenotypic correlation between traits is moderately high, we can predict the information of thousands of lines using cross-validation CV2 (in this case, 9091 lines were predicted for year_14_15, 9501 lines for year_15_16, and 9917 lines for year_17_17). The IBCF technique also has the advantage that the implementation time is fast and allows parallel computing in the event of larger data sets.

A disadvantage of using IBCF is that it works with phenotypic correlations between items (trait–environment combinations) or users (genotypes), and when we use the genomic relationship matrix as a proxy for the phenotypic correlation between genotypes, this does not really represent the phenotypic correlation between genotypes and can produce poor predictions. This was observed in our results where only in the second real data set using the genomic relationship matrix produced a little improvement in prediction accuracy when the genomic relationship matrix was used. When applied to the first real data set (wheat data set), the predictions obtained were poor. Also, it is important to point out that due to the nature of both models (IBCF and matrix factorization), they not allow for inclusion of the genomic relationship matrix. For this reason, we used the genomic relationship matrix as a proxy for the phenotypic correlation between genotypes (users) to be able to implement the UBCF technique, but we did not find an efficient way to incorporate this information under the matrix factorization method. However, according to the results obtained, we have evidence that using the genomic relationship matrix alone as proxy for the phenotypic correlation between genotypes (users) using the UBCF is not reasonable. For this reason, we believe that to take into account the genomic relationship matrix in these models (IBCF and matrix factorization), some novel modifications need to be made to these recommender systems methods to be able to take advantage of all the genomic information.

We must point out that the process for scaling each column is very important in order to have all the columns (items = trait–environment combinations) on the same scale, since methods IBCF and matrix factorization were originally proposed for ordinal items (example 1 = totally disagree, 2 = disagree, 3 = neutral, 4 = agree, 5 = totally agree). For this reason, the user must remember to scale each column of the rating matrix. We encourage the use of both IBCF and matrix factorization for ordinal or binary phenotypic data, given that IBCF was originally proposed in the context of ordinal data. However, according to our review of these two statistical techniques (methods IBCF and matrix factorization), they had only been implemented using the cosine and Pearson correlation. However, we believe that predictions can be improved using the polychoric correlation for ordinal data or the tetrachoric correlation for binary data. Also, since we usually use 0, 1, or 2 to denote the information of each marker in a line in its position in the genome with SNPs, we believe that when the correlation between the columns of SNPs is reasonably large, this method can be used successfully for imputation of missing markers.

It is important to point out that both methods can be implemented using the R code given in Appendices A1 and A2. Although there are some R packages that can be used to implement IBCF and matrix factorization, we need to be careful when using these packages, since some of them were developed exclusively for binary or ordinal data; thus, when we want to use them for scaled continuous data, they do not work because the maximization process they use is only for positive values of the input provided. One package that has such restrictions is the package rrecsys (Coba *et al.* 2017).

Finally, one of the main advantages of using IBCF is that its implementation is very fast and can be used on large data sets without problems. In the case of data sets with very large numbers of columns, parallel processing is possible because its computation is not iterative. However, if the correlation between the columns of the created rating matrix is poor (low correlation), this technique produces poor predictions; the larger the correlation between the columns of the rating matrix, the better the sample predictions will be.

### Conclusions

It is necessary to improve the accuracy of the prediction models used in GS. For this reason, we explored two recommender systems techniques: IBCF and matrix factorization. Both are very popular in the context of online marketing to recommend products or items. The IBCF technique is attributed to Amazon.com (Linden *et al.* 2003), which implemented it as its recommender system. It works based on the similarity between items, calculated using people’s ratings of those items (Sarwar *et al.* 2001). The IBCF uses the items most similar to a user’s already rated items to generate a list of predictions (recommendations). Usually the predictions are a weighted sum or linear regression. From our results using real and simulated data, we obtained empirical evidence showing that both methods, IBCF and matrix factorization, work well for predicting phenotypes that are missing in some traits and environments, but the IBCF was the best. Both methods are very efficient if, and only if, the correlation between traits and between environments is moderately high. However, when the correlation between traits and between environments is low, the performance of both techniques in terms of prediction accuracy is poor. We believe that more empirical evidence is required to be able to consider these techniques as an attractive alternative for whole-genome prediction. Additionally, we observed that the implementation of the IBCF technique is fast and can be used for large data sets. It produced reasonable predictions of thousands of lines for a trait that needs to be predicted next year.

## Appendix A1

### R Code for Implementing Item-Based Collaborative Filtering

rec_IBCF=function(geno_no)

{ genoRatings=ratings[geno_no,]

non_rated_items=list()

rated_items=list()

for(i in 2:ncol(genoRatings)){

if(is.na(genoRatings[,i]))

{non_rated_items=c(non_rated_items,colnames(genoRatings)[i])}

else

{rated_items=c(rated_items,colnames(genoRatings)[i])}}

non_rated_items = unlist(non_rated_items)

rated_items = unlist(rated_items)

#create weighted similarity for all the rated items by geno

non_rated_pred_score = list()

for(j in 1:length(non_rated_items)){

temp_sum=0

df =item_sim[which(rownames(item_sim)==non_rated_items[j]),]

for(i in 1:length(rated_items)){

temp_sum = temp_sum+ abs(df[which(names(df)==rated_items[i])])}

weight_mat = df*ratings[geno_no,2:(length(genoRatings))]

non_rated_pred_score = c(non_rated_pred_score,rowSums(weight_mat,na.rm=T)/temp_sum)}

pred_rat_mat = as.data.frame(non_rated_pred_score)

names(pred_rat_mat) = non_rated_items

for(k in 1:ncol(pred_rat_mat)){

ratings[geno_no,][which(names(ratings[geno_no,]) == names(pred_rat_mat)[k])]=pred_rat_mat[1,k]}

return(ratings[geno_no,])}

## Appendix A2

### R Code for Implementing the Matrix-Factorization Algorithm with Regularization

rec_Mat_fact<-function(R,P,Q,K,maxiter,alpha,lambda,tol)

{

Q=t(Q)

for(s in 1:maxiter)

{for(i in 1:dim(R)[1]) {for(j in 1:dim(R)[2]){

if(!is.na(R[i,j]))

{e_ij = R[i,j] - crossprod(P[i,],Q[,j])

for(k in 1:K)

{

P[i,k] = P[i,k] + alpha * (2 * e_ij * Q[k,j] - lambda * P[i,k])

Q[k,j] = Q[k,j] + alpha * (2 * e_ij * P[i,k] - lambda * Q[k,j])

}}}}

sse=0

for(i in 1:dim(R)[1])

{for(j in 1:dim(R)[2]){

if(!is.na(R[i,j])){

sse = sse + (R[i,j] - crossprod(P[i,],Q[,j]))^2

for(k in 1:K)

{sse = sse + lambda/2*(P[i,k]^2+Q[k,j]^2)}}}}

if(sse<tol)

break }

list(P=P,Q=t(Q))}

## Appendix A3

### Example for Implementing the IBCF and Matrix-Factorization Algorithms

library(mvtnorm)

library(lsa)

########Generating data from 15 lines (users) and traits (items) ##############

set.seed(2)

A=matrix(0.85,ncol=5,nrow=5)

diag(A)=1

R=rmvnorm(15,mean=rep(0,5),sigma=A)

########Selecting 20% as missing data######################################

pos.missing=sample(1:75,15,replace=F)

pos.missing

R_mis=R

R_mis[pos.missing]=NA

############Loading the IBCF and the Matrix factorization methods###############

source(“Colab_Filtering_IBCF.r”) ###Code given in Appendix A1

source(“Colab_Filtering_Mat_Fact_Final.r”) ###Code given in Appendix A2

############Predictions using the IBCF method#############################

ratings=data.frame(cbind(c(1:nrow(R_mis)),R_mis))

colnames(ratings)=c(“geno_no”,”I1”, “I2”, “I3”, “I4”, “I5”)

ratings

nrow_ratings=nrow(ratings)

ncol_ratings=ncol(ratings)

x=R_mis

x[is.na(x)] = 0

item_sim=cosine(as.matrix((x)))

colnames(item_sim)=c(“I1”, “I2”, “I3”, “I4”, “I5”)

rownames(item_sim)=c(“I1”, “I2”, “I3”, “I4”, “I5”)

item_sim

R.pred=data.matrix(ratings)

##############Row positions with no missing values########################

pos.wn.Na=c(as.numeric(noquote(rownames(na.omit(data.frame(ratings))))))

R.pred=data.matrix(ratings)

##############Row positions with no missing values########################

pos.wn.Na=c(as.numeric(noquote(rownames(na.omit(data.frame(ratings))))))

###############All rows to use###########################################

pos.used=c(1:nrow_ratings)

##############Row positions with missing values########################

pos.final=pos.used[-pos.wn.Na]

for (i in 1:length(pos.final))

{

pos=pos.final[i]

R.pred[pos,c(2:ncol_ratings)]=c(data.matrix(rec_IBCF((pos))))[2:ncol_ratings]

}

R.pred1=R.pred[,-1]

#R.pred1[pos.missing]

#R.pred_IBCF

setwd(“C:\\Multi-trait analysis Crossa”)

library(mvtnorm)

library(lsa)

########Generating data from 15 lines (users) and traits (items) ##############

A=matrix(0.85,ncol=5,nrow=5)

diag(A)=1

R=rmvnorm(15,mean=rep(0,5),sigma=A)

R

########Selecting 20% as missing data######################################

pos.missing=sample(1:75,15,replace=F)

pos.missing

R_mis=R

R_mis[pos.missing]=NA

R_mis

############Loading the IBCF and the Matrix factorization methods###############

source(“Colab_Filtering_IBCF.r”) ###Code given in Appendix A1

source(“Colab_Filtering_Mat_Fact_Final.r”) ###Code given in Appendix A2

############Predictions using the IBCF method###############################

ratings=data.frame(cbind(c(1:nrow(R_mis)),R_mis))

colnames(ratings)=c(“geno_no”,”I1”, “I2”, “I3”, “I4”, “I5”)

ratings

nrow_ratings=nrow(ratings)

ncol_ratings=ncol(ratings)

x=R_mis

x[is.na(x)] = 0

item_sim=cosine(as.matrix((x)))

colnames(item_sim)=c(“I1”, “I2”, “I3”, “I4”, “I5”)

rownames(item_sim)=c(“I1”, “I2”, “I3”, “I4”, “I5”)

item_sim

R.pred=data.matrix(ratings)

##############Row positions with no missing values########################

pos.wn.Na=c(as.numeric(noquote(rownames(na.omit(data.frame(ratings))))))

pos.wn.Na

###############All rows to use############################################

pos.used=c(1:nrow_ratings)

##############Row positions with missing values########################

pos.final=pos.used[-pos.wn.Na]

for (i in 1:length(pos.final))

{

pos=pos.final[i]

R.pred[pos,c(2:ncol_ratings)]=c(data.matrix(rec_IBCF((pos))))[2:ncol_ratings]

}

R.pred_IBCF=R.pred[,-1]

R.pred_IBCF

############Predictions using the matrix factorization method (MF)##############

K = 3 #####Number of latent features

n = dim(R_mis)[1]

L = dim(R_mis)[2]

P = matrix(rnorm(n*K,0,1),nc=K) ####Starting values of P

Q = matrix(rnorm(L*K,0,1),nc=K) ####Starting values of Q

Mat_fact=rec_Mat_fact(R=R_mis,P,Q,K,maxiter=1e3,alpha = 2e-3,lambda=21e-3,tol=1e-2)

#Predictions

output=Mat_fact$P%*%t(Mat_fact$Q)

R_pred_Mat_Fact=R_mis

R_pred_Mat_Fact[pos.missing]=output[pos.missing]

R_pred_Mat_Fact

## Appendix A4

### Phenotypic cosine correlations for environment-trait combinations in data set 1 (wheat data set)

**Table:**

Environments	Bed5IR_ HD	Bed5IR_ GNDVI	Bed5IR_ GY	Bed5IR_ PH	Bed2IR_ HD	Bed2IR_ GNDVI	Bed2IR_ GY	Bed2IR_ PH	Drip_ HD	Drip_G_ GNDVI	Drip_ GY	Drip_ PH
Bed5IR_HD	1.000	0.698	−0.271	−0.120	0.564	0.461	0.207	0.341	0.486	0.192	−0.036	−0.223
Bed5IR_GNDVI	0.698	1.000	−0.204	−0.035	0.470	0.527	0.131	0.259	0.398	0.286	−0.032	−0.148
Bed5IR_GY	−0.271	−0.204	1.000	0.577	−0.149	−0.058	0.207	0.138	−0.324	−0.385	0.481	0.187
Bed5IR_PH	−0.120	−0.035	0.577	1.000	−0.052	0.083	0.247	0.318	−0.157	−0.262	0.397	0.236
Bed2IR_HD	0.564	0.470	−0.149	−0.052	1.000	0.787	0.386	0.628	0.505	0.201	−0.020	−0.268
Bed2IR_GNDVI	0.461	0.527	−0.058	0.083	0.787	1.000	0.493	0.640	0.401	0.166	0.100	−0.144
Bed2IR_GY	0.207	0.131	0.207	0.247	0.386	0.493	1.000	0.534	0.147	−0.034	0.192	−0.085
Bed2IR_PH	0.341	0.259	0.138	0.318	0.628	0.640	0.534	1.000	0.213	−0.089	0.277	0.013
Drip_HD	0.486	0.398	−0.324	−0.157	0.505	0.401	0.147	0.213	1.000	0.551	−0.401	−0.535
Drip_G_NDVI	0.192	0.286	−0.385	−0.262	0.201	0.166	−0.034	−0.089	0.551	1.000	−0.575	−0.298
Drip_GY	−0.036	−0.032	0.481	0.397	−0.020	0.100	0.192	0.277	−0.401	−0.575	1.000	0.460
Drip_PH	−0.223	−0.148	0.187	0.236	−0.268	−0.144	−0.085	0.013	−0.535	−0.298	0.460	1.000

## Appendix A5

### Phenotypic cosine correlations for environment-trait combinations in data set 2 (maize data set)

**Table:**

	EBU_GY	EBU_ASI	EBU_PH	KAK_GY	KAK_ASI	KAK_PH	KTI_GY	KTI_ASI	KTI_PH
EBU_GY	1.000	−0.146	0.197	0.055	−0.029	0.236	0.120	−0.049	0.107
EBU_ASI	−0.146	1.000	−0.134	0.097	0.133	0.049	−0.013	0.045	−0.057
EBU_PH	0.197	−0.134	1.000	−0.094	0.100	0.068	0.043	0.034	0.010
KAK_GY	0.055	0.097	−0.094	1.000	−0.078	0.351	0.110	0.047	−0.013
KAK_ASI	−0.029	0.133	0.100	−0.078	1.000	−0.138	0.020	0.188	−0.036
KAK_PH	0.236	0.049	0.068	0.351	−0.138	1.000	0.016	0.057	0.080
KTI_GY	0.120	−0.013	0.043	0.110	0.020	0.016	1.000	−0.263	0.429
KTI_ASI	−0.049	0.045	0.034	0.047	0.188	0.057	−0.263	1.000	−0.318
KTI_PH	0.107	−0.057	0.010	−0.013	−0.036	0.080	0.429	−0.318	1.000

## Appendix A6

### Phenotypic cosine correlations for environment-trait combinations in data set 3 (large wheat data set)

**Table:**

	HD	DMT	PH	Lodging	GY
HD	1.000	0.760	0.228	−0.069	0.401
DMT	0.760	1.000	0.251	0.012	0.454
PH	0.228	0.251	1.000	−0.165	0.386
Lodging	−0.069	0.012	−0.165	1.000	−0.443
GY	0.401	0.454	0.386	−0.443	1.000

## Appendix A7

### Simulated data

**Table:**

		Cor = 0.85				Cor = 0.5				Cor = 0.25			
Scenario	Env–Trait	IBCF	MF	ME	MTME	IBCF	MF	ME	MTME	IBCF	MF	ME	MTME
	E1_T1	0.004	0.005	0.034	0.007	0.008	0.008	0.039	0.007	0.010	0.010	0.042	0.008
	E2_T1	0.004	0.006	0.024	0.010	0.008	0.009	0.041	0.011	0.009	0.011	0.045	0.010
	E3_T1	0.005	0.007	0.021	0.008	0.008	0.010	0.041	0.009	0.010	0.012	0.049	0.009
S1	E1_T2	0.005	0.006	0.014	0.008	0.010	0.010	0.026	0.008	0.012	0.012	0.031	0.008
	E2_T2	0.005	0.006	0.036	0.010	0.009	0.009	0.038	0.011	0.010	0.011	0.038	0.012
	E3_T2	0.005	0.006	0.021	0.008	0.009	0.009	0.034	0.010	0.010	0.010	0.038	0.011
	E1_T3	0.004	0.005	0.017	0.009	0.006	0.006	0.036	0.009	0.007	0.007	0.046	0.010
	E2_T3	0.004	0.004	0.023	0.010	0.009	0.008	0.050	0.010	0.011	0.011	0.060	0.010
	E3_T3	0.004	0.006	0.029	0.008	0.008	0.009	0.035	0.010	0.010	0.010	0.038	0.011
	Average	0.004	0.006	0.024	0.009	0.008	0.009	0.038	0.009	0.010	0.010	0.043	0.010
	E1_T1	0.006	0.006	0.009	0.002	0.011	0.011	0.014	0.003	0.013	0.013	0.018	0.004
	E2_T1	0.003	0.004	0.014	0.003	0.006	0.007	0.031	0.005	0.008	0.009	0.041	0.006
	E3_T1	0.005	0.005	0.018	0.002	0.007	0.008	0.034	0.003	0.008	0.009	0.038	0.003
S2	E1_T2	0.006	0.007	0.021	0.003	0.010	0.010	0.040	0.004	0.011	0.011	0.045	0.005
	E2_T2	0.004	0.004	0.014	0.003	0.007	0.007	0.025	0.005	0.008	0.009	0.031	0.007
	E3_T2	0.006	0.006	0.023	0.002	0.010	0.010	0.047	0.005	0.011	0.011	0.052	0.006
	E1_T3	0.004	0.005	0.019	0.003	0.008	0.008	0.040	0.004	0.008	0.009	0.045	0.005
	E2_T3	0.005	0.005	0.014	0.005	0.009	0.009	0.031	0.007	0.010	0.010	0.034	0.008
	E3_T3	0.005	0.005	0.013	0.003	0.010	0.010	0.021	0.005	0.011	0.011	0.025	0.006
	Average	0.005	0.005	0.016	0.003	0.009	0.009	0.031	0.005	0.010	0.010	0.037	0.006
	E1_T1	0.003	0.004	0.040	0.009	0.008	0.008	0.046	0.009	0.010	0.010	0.049	0.010
	E2_T1	0.003	0.003	0.051	0.008	0.006	0.006	0.068	0.008	0.007	0.007	0.067	0.009
	E3_T1	0.003	0.004	0.040	0.012	0.007	0.007	0.050	0.014	0.009	0.009	0.051	0.014
	E1_T2	0.004	0.004	0.043	0.011	0.007	0.007	0.060	0.010	0.009	0.009	0.056	0.010
S3	E2_T2	0.004	0.004	0.059	0.014	0.007	0.007	0.057	0.014	0.008	0.008	0.062	0.012
	E3_T2	0.005	0.005	0.040	0.015	0.009	0.009	0.048	0.012	0.011	0.011	0.050	0.011
	E1_T3	0.005	0.005	0.034	0.010	0.009	0.009	0.039	0.009	0.011	0.011	0.046	0.008
	E2_T3	0.004	0.004	0.049	0.012	0.009	0.009	0.037	0.011	0.012	0.011	0.049	0.011
	E3_T3	0.005	0.005	0.053	0.012	0.010	0.010	0.044	0.011	0.011	0.011	0.039	0.010
	Average	0.004	0.004	0.045	0.012	0.008	0.008	0.050	0.011	0.010	0.010	0.052	0.011

SE of the prediction accuracies given in Figure 1, Figure 2, and Figure 3, with Pearson correlation for each environment–trait (Env–Trait) combination using the four methods under study [IBCF, matrix factorization (MF), ME, and MTME] for data simulated with a correlation (Cor) of traits (genetic and residual) and correlation of environments of 0.85, 0.5, and 0.25. S1 is the scenario under normality, S2 is the scenario under the error negative skew multiplied by 1.25, and S3 represents the scenario under the error positive skew multiplied by 1.25. The notation E1_T1 means environment 1, trait 1.
